# Magnetic resonance imaging radiomics to differentiate ovarian sex cord-stromal tumors and primary epithelial ovarian cancers

**DOI:** 10.3389/fonc.2022.1073983

**Published:** 2023-01-13

**Authors:** Meiying Cheng, Shifang Tan, Tian Ren, Zitao Zhu, Kaiyu Wang, Lingjie Zhang, Lingsong Meng, Xuhong Yang, Teng Pan, Zhexuan Yang, Xin Zhao

**Affiliations:** ^1^Department of Radiology, Third Affiliated Hospital of Zhengzhou University, Zhengzhou, Henan, China; ^2^Department of Information, Third Affiliated Hospital of Zhengzhou University, Zhengzhou, Henan, China; ^3^Medical College, Wuhan University, Wuhan, China; ^4^Magnetic resonance imaging (MRI) Research, GE Healthcare (China), Beijing, China; ^5^Department of Research, Huiying Medical Technology Co., Ltd., Beijing, China; ^6^Department of Research, Third Affiliated Hospital of Zhengzhou University, Zhengzhou, Beijing, China

**Keywords:** ovarian sex cord-stromal tumor, epithelial ovarian cancer, magnetic resonance imaging, radiomics, prediction model

## Abstract

**Objective:**

To evaluate the diagnostic ability of magnetic resonance imaging (MRI) based radiomics and traditional characteristics to differentiate between Ovarian sex cord-stromal tumors (SCSTs) and epithelial ovarian cancers (EOCs).

**Methods:**

We consecutively included a total of 148 patients with 173 tumors (81 SCSTs in 73 patients and 92 EOCs in 75 patients), who were randomly divided into development and testing cohorts at a ratio of 8:2. Radiomics features were extracted from each tumor, 5-fold cross-validation was conducted for the selection of stable features based on development cohort, and we built radiomics model based on these selected features. Univariate and multivariate analyses were used to identify the independent predictors in clinical features and conventional MR parameters for differentiating SCSTs and EOCs. And nomogram was used to visualized the ultimately predictive models. All models were constructed based on the logistic regression (LR) classifier. The performance of each model was evaluated by the receiver operating characteristic (ROC) curve. Calibration and decision curves analysis (DCA) were used to evaluate the performance of models.

**Results:**

The final radiomics model was constructed by nine radiomics features, which exhibited superior predictive ability with AUCs of 0.915 (95%CI: 0.869-0.962) and 0.867 (95%CI: 0.732-1.000) in the development and testing cohorts, respectively. The mixed model which combining the radiomics signatures and traditional parameters achieved the best performance, with AUCs of 0.934 (95%CI: 0.892-0.976) and 0.875 (95%CI: 0.743-1.000) in the development and testing cohorts, respectively.

**Conclusion:**

We believe that the radiomics approach could be a more objective and accurate way to distinguish between SCSTs and EOCs, and the mixed model developed in our study could provide a comprehensive, effective method for clinicians to develop an appropriate management strategy.

## Introduction

Ovarian sex cord-stromal tumors (SCSTs) are rare nonepithelial neoplasms, and represent about 7% of all primary ovarian tumors (1, 2). According to the classification of ovarian tumors by World Health Organization (WHO) (2020), SCSTs are divided into the following three clinicopathologic subcategories: pure stromal tumors, pure sex cord tumors, and mixed sex cord-stromal tumors (3). Fibromas, thecomas, and granulosa cell tumors account for the majority of SCSTs.

Morphologically, SCSTs usually present as solid masses (4), resembling malignant tumors. Although some of the SCSTs, such as fibromas, have a few specific characteristics on conventional imaging features, diagnostic dilemmas may often arise if the tumor shows increased cellularity, or due to necrosis, hemorrhage, edema, or cystic degeneration (2, 4). Other types are even more confusing on account of morphological complexity. Moreover, the rarity of SCSTs contributes to a low degree of suspicion, which makes it susceptible to misdiagnosis as the more common epithelial ovarian cancer (EOC). Clinically, SCSTs most commonly present at early stages (I) and are primarily surgically treated with an overall favorable prognosis (2, 5), while epithelial tumors usually present at advanced stages (III or IV) and are treated with chemotherapy and surgical debulking (2). Hence, a more accurate and objective assessment method to identify SCSTs from ovarian cancers is imperative.

Magnetic resonance imaging (MRI) has been widely used to detect and evaluate adnexal lesions, especially for the indeterminate adnexal masses on ultrasonography (6, 7). The high soft-tissue resolution and ability to characterize the composition of different fluid types allow it to characterize the lesion types more accurately (7). However, all the evaluation by MRI requires the subjective interpretation of radiologists, in addition to the complexity and overlap in imaging characteristics of different diseases, it is still a challenge to differentiate the types of tumors with MRI alone (6).

Radiomics is an emerging method for postprocessing any type of medical image and generating new quantification metrics which have proven to provide important insights into tumor biology, shifting radiology from the traditional visual analysis to a more objective and automated analysis (6). Recently, radiomics studies on adnexal tumors have demonstrated some encouraging advances, including clinical outcomes in ovarian cancer (6, 8, 9), tumor category (9, 10), and subtype differentiation (9, 11). However, there are few studies involving SCSTs. In this study, we developed an MR image-based radiomics diagnostic model to prove that SCSTs and EOCs are separable.

## Patients and methods

### Patient population

**Figure 1:**
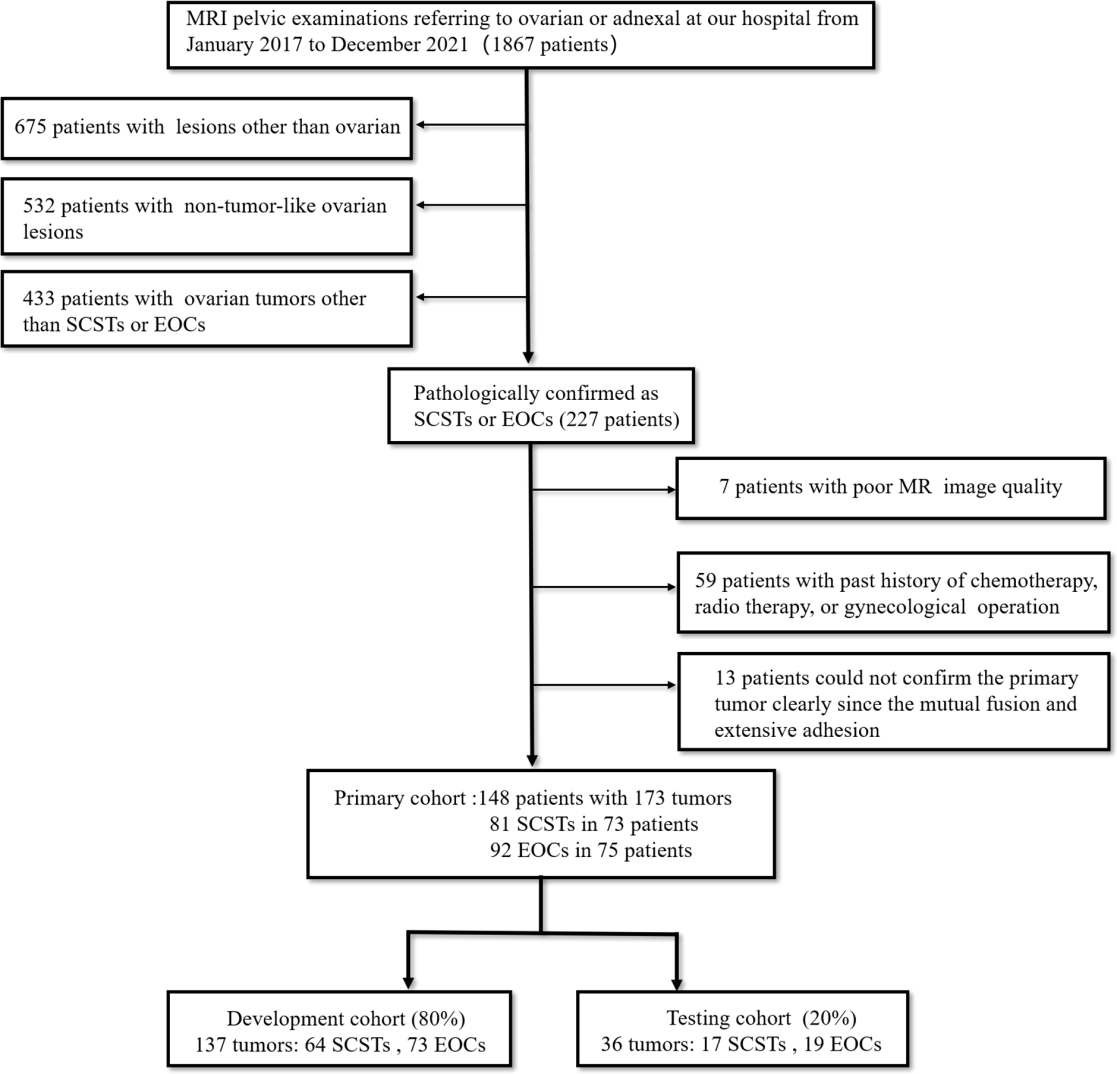
Flowchart for the patient selection process in this study.

This retrospective pilot study was approved by our Institutional Review Board with a waiver of informed consent. From January 2017 to December 2021, we retrospectively retrieved all MRI pelvic examinations referring to ovarian or adnexal lesions from our Institutional Picture Archiving and Communication System (PACS) and obtained 1867 results. Every report was analyzed by the researchers. Then we identified patients who met the following inclusion criteria (1): pathologically confirmed as SCSTs or EOCs; (2) MRI was performed within 1 month before pelvic or laparoscopic surgery at our institution; (3) no chemotherapy, radiotherapy, or previous gynecological operation prior to MRI examination. The exclusion criteria were: (1) poor MR image quality, so that the focus cannot be clearly observed or delineated; (2) multiple nodules that the primary focus cannot be identified due to a mutual fusion and extensive adhesion pattern or any other reason. Finally, we consecutively reviewed a total of 148 patients with 173 tumors (81 SCSTs in 73 patients and 92 EOCs in 75 patients) as the primary cohort. Details of histopathology are presented in **Table 1**. Then the primary dataset was randomly split into the development and testing cohorts with a fixed ratio of 8:2 in each category, resulting in 137 tumors (64 SCSTs and 73 EOCs) for the development cohort and 36 tumors (17 SCSTs and 19 EOCs) for testing cohort. A flowchart of the patient selection process is shown in **Figure 1**.

**Table 1:** Histopathological types of the selected samples.

Pathological type	Numbers
SCST	81
Thecoma	4
Fibroma	18
Cellular fibroma	2
Fibrothecoma	28
Granulosa cell tumor	25
Sclerosing stromal tumor	2
Sertoli-Leydig cell tumor	2
EOC	92
High grade serous carcinoma	42
Low grade serous carcinoma	17
Endometrioid carcinoma	12
Clear cell carcinoma	12
Mucinous carcinoma	6
Seromucinous carcinoma	3
Total	173

SCST, sex cord-stromal tumors; EOC, epithelial ovarian cancer

Clinical characteristics such as patient age, menstrual status, endocrine level, cancer antigen 125 (CA125), and risk of ovarian malignancy algorithm (ROMA), were obtained from the hospital information system.

### MR image acquisition and interpretation

MR examinations were performed on the 3.0 T system (SIGNA Pioneer, GE Healthcare, and Skyra, Siemens Healthcare) with the phased-array abdominal coil. The conventional MR sequences included T1 weighted imaging (T1WI) in the axial plane, T2 weighted imaging (T2WI) in the axial and sagittal plane, fat-suppressed T2WI (FS-T2WI) in the axial and coronal plane, diffusion-weighted imaging (DWI) with the b value of 1000 s/mm^2^ in the axial plane, and multiphase contrast-enhanced fat-suppressed T1WI in the axial, sagittal, and coronal plane. Detailed information about the acquisition parameters is presented in **Table S1**.

Two radiologists (reader 1 and reader 2, with 7 and 15 years of experience in MRI, respectively) independently recorded the conventional imaging features while blinded to the histological results. The recorded features include (1) Maximum diameter (MD) (measured at the lesion slice with the maximum diameter of the tumor in the three-dimensional measurements); (2) Visibility of hemorrhagic component; (3) Solid and cystic components (predominantly cystic, cystic-solid, and predominantly solid corresponding to less than 1/3, 1/3-2/3, and more than 2/3 solid component, respectively); (4) Signal intensity (SI) of the solid components on T2WI (Hypo-, iso-, or hyperintense was relative to the external myometrium; A few purely cystic lesions were not recorded); (5) Apparent diffusion coefficient (ADC) value (measured manually on the DWI derived ADC maps; regions of interest were placed at target areas of the tumor, and areas such as necrosis, hemorrhage, vascular structures were avoided as much as possible; three measurements were obtained and averaged). Discrepancies were resolved by a consensus, or a third radiologist (reader 3 with more than 20 years of experience in gynecologic imaging) would serve as an arbitrator.

### Tumor segmentation

In order to reduce the discrepancies related to various scanning parameters and eliminate the internal dependence of image radiomics features on voxel size, all images were set to a fixed voxel size (1mm×1mm×1mm) with linear interpolation algorithm.

The 3D segmentation was performed on open-source software (ITK-SNAP version 3.8.0, http://www.itksnap.org). The volume of interest (VOI) for each lesion on each slice was manually delineated on FS-T2WI by a reader 1. Then, reader 3 confirmed all the regions. To examine the reproducibility of extracted radiomics features and to obtain more robust radiomics features, the intra-class correlation coefficient (ICC) was used to assess the intra- and inter-observer agreement of VOI delineation. So, four weeks later, 30 patients (15 SCSTs and 15 EOCs) were randomly selected. Reader 1 then re-delineated the VOI for intra-consistency testing, in the meantime, reader 2 outlined the VOI according to the same procedure to test inter-consistency. The feature with an ICC > 0.75 was selected for further analysis (12). ICC can be obtained from the following equation:


, $$ICC=\frac{\left(MS_{R}-MS_{E}\right)}{MS_{R}+(\frac{MS_{C}-MS_{E}}{n})}$$

where MS_R_ is mean square for rows, MS_C_ is the mean square for columns, MS_E_ is mean square for error and n represents the number of subjects.

### Feature extraction

The extraction of radiomics features was conducted in the Radcloud software (Huiying Medical Technology Co., Ltd, Beijing, China). A total of 1,409 features were extracted from the FS-T2WI sequences using the pyradiomics function package (https://pyradiomics.readthedocs.io/). These features could be divided into the following three categories. First, first-order statistical features, such as peak value, mean, and variance, quantitatively describe the voxel intensity distribution of the lesion area in MR images through common basic indicators. Second, two-dimensional morphological features, describe the two-dimensional shape and size of the lesion. Third, texture features, such as Gray Level Co-occurrence Matrix (GLCM), Gray Level Run Length Matrix (GLCM), and Gray Level Size Zone Matrix (GLSZM), quantify the heterogeneity of the lesion texture. In addition, several filters, such as exponential, logarithm, square, square root, and wavelet (including wavelet-LHL, wavelet-LHH, wavelet-HLL, wavelet-LLH, wavelet-HLH, wavelet-HHH, wavelet-HHL, and wavelet-LLL) filters, were applied to calculate the first-order statistical features and texture features of the transformed image.

### Feature selection

Before the selection of radiomics features, normalization processing was performed for all extracted features, and the features were normalized to the normal distribution by mean and variance scaling.

We run a 5-fold cross validation to select features, where each fold we did feature selection and model building on 80% of the development data (the training cohort), and evaluated on the remaining 20% (the validation cohort). In each fold, we implement the dimensionality reduction process in three steps. Features with a variance value of >0.8 were first selected. Then SelectKBest was applied to select the features with a *p*-value less than 0.05. Finally, the Lassolars algorithm was used to screen the optimal radiomics features, among which the top 10 features with the best contribution would be selected. The least absolute shrinkage and selection operator (LASSO) is a regression analysis method that can perform both variable selection and regularization to improve the identification accuracy and interpretability of the model. Regression algorithm least angle regression (LARS) provides variables through the linear combination of high-dimensional data. It is related to positive stepwise regression. Lassolars algorithm used in this paper is a combination of Lars algorithm and lasso model, which can automatically select the optimal parameters λ and have better performance than LASSO algorithm alone (13). Finally, we counted the frequency of each feature selected in the 5 folds, the features that get selected three or more times repeatedly were considered stable features, and different models were built using various combinations of those selected features.

### Classifier modeling

**Figure 2:**
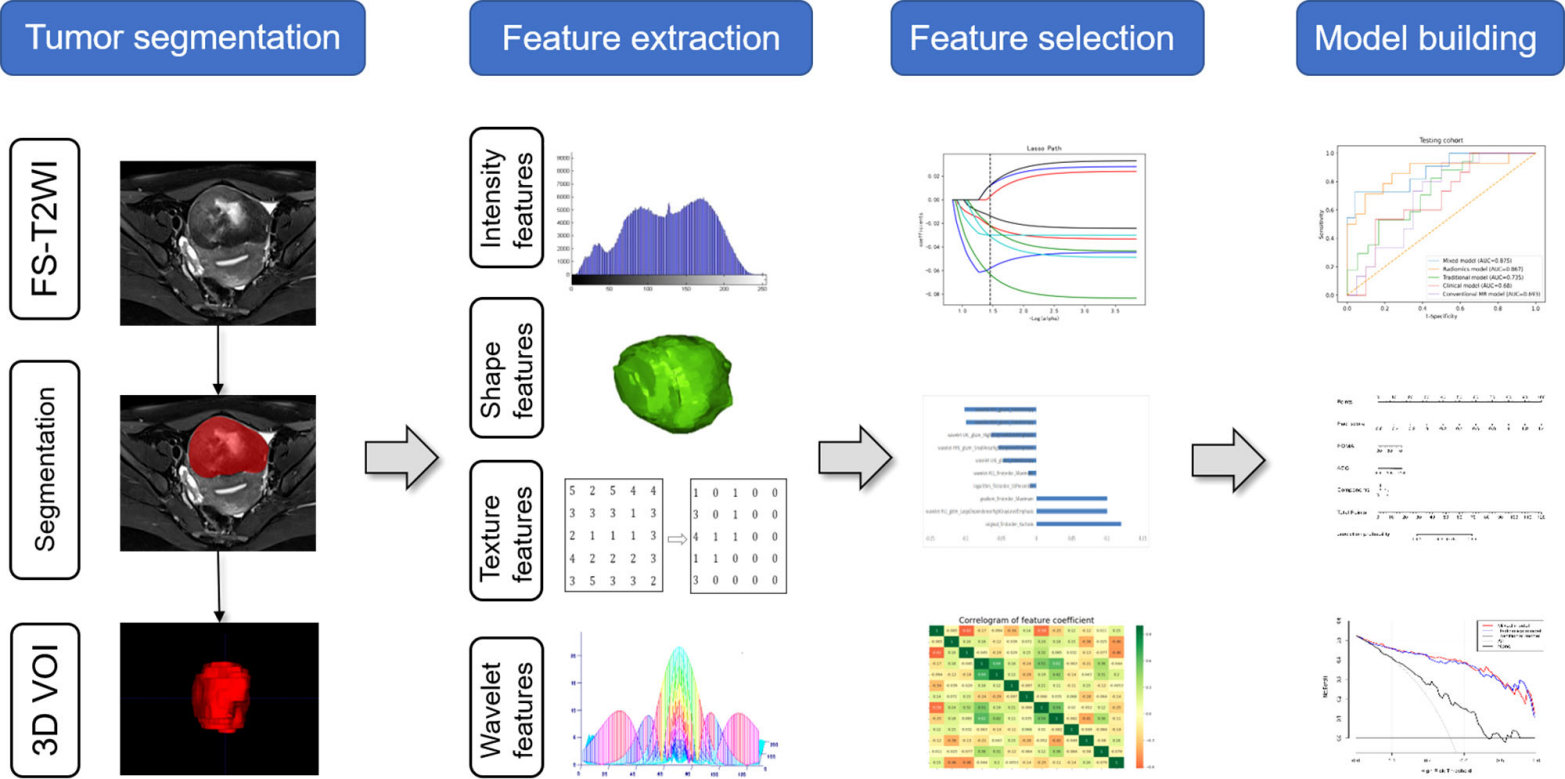
Process of radiomics analysis in this study.

Univariate analysis and multivariate logistic regression analysis were successively performed based on development data to screen the clinical and conventional MR features. Based on the selected radiomics features, clinical features, and conventional MR parameters, five prediction models (clinical model, conventional MR model, traditional model, radiomics model and mixed model) were constructed by using the LR classifier. LR classifier includes classification, function establishment, solving optimal model parameters through optimization iteration, and verifying the model performance. In addition, the nomogram was also constructed to visualize the results of the logistic regression. The nomogram develops scoring criteria based on the magnitude of the regression coefficients for all independent variables. It scores each value level of each independent variable, giving an overall score and finally calculating the probability of disease risk for each patient through a conversion function between the score and the outcome probability. The calibration curve was drawn to assess the agreement between the predicted results and actual presence. The detailed process of radiomics analysis is presented in **Figure 2**.

The performance of the model was estimated by using receiver operating characteristic (ROC) curves and confusion matrix analysis, and the area under the curve (AUC), sensitivity, specificity, and accuracy were calculated. The DeLong test was used to compare the performance of different models. The decision curve analysis (DCA) was used to evaluate the clinical utility of the prediction models by quantifying the net benefits at different threshold probabilities in the dataset.

### Statistical analysis

Statistical analysis was performed using SPSS version 25.0 (IBM). Quantitative variables are shown as mean ± standard deviation. Categorical variables were assessed by Chi-square tests or Fisher exact test, and differences in continuous variables were assessed by t-test or Mann-Whitney U test. *P*< 0.05 was considered statistically significant. Model construction was executed using R software 3.5.3 (https://cran.r-project.org/) and Python 2.7 software (https://www.python.org/). The packages of “pyradiomics” (https://pyradiomics.readthedocs.io/), “scikitlearn” (https://scikit-learn.org/), and “matplotlib” (https://matplotlib.org/) were used for feature selection, model building, and plotting in this study.

## Results

### Patients and tumor characteristics

The comparisons of the clinical data and MR parameters between SCSTs and EOCs groups in the primary and development cohorts are summarized in **Table 2**. Age, ROMA index, serum CA125, ADC value, MD, SI on T2WI, solid and cystic components showed significant differences between the SCSTs and EOCs groups, while no significant difference was observed in menstrual status, endocrine level, and hemorrhagic component. Among the variables with significant differences, only ROMA index (*P*< 0.001), ADC value (*P* = 0.004), solid and cystic components (*P* = 0.043) remained as independent predictors on the multivariate logistic regression analysis.

**Table 2:** The clinical data and MR parameters of the primary and development cohorts.

Characteristics	Primary cohort			Development cohort		
	EOCs (n = 92)	SCSTs (n = 81)	*P* value	EOCs (n = 73)	SCSTs (n = 64)	*P* value
Age (year) ^a^	51.78 ± 10.35	46.33 ± 15.76	0.017^*^	52.22 ± 10.41	46.58 ± 16.16	0.015^*^
ROMA (%) ^a^	32.07 ± 26.71	16.03 ± 12.20	<.001^*^	33.65 ± 27.32	16.08 ± 12.91	<.001^*^
CA125 (μg/L) ^b^			<.001^*^			<.001^*^
<35	38 (41.3%)	55 (67.9%)		27 (37.0%)	46 (71.9%)	
35-200	26 (28.3%)	23 (28.4%)		23 (31.5%)	15 (23.4%)	
200-500	13 (14.1%)	1 (1.2%)		10 (13.7%)	1 (1.6%)	
>500	15 (16.3%)	2 (2.5%)		13 (17.8%)	2 (3.1%)	
Menstrual status ^b^			0.543			0.205
Premenopausal	45 (48.9%)	44 (54.3%)		32 (43.8%)	35 (54.7%)	
Postmenopausal	47 (51.1%)	37 (45.7%)		41 (56.2%)	29 (45.3%)	
Endocrine level ^b^			0.788			0.588
Normal	85 (92.4%)	73 (90.1%)		67 (91.8%)	57 (89.1%)	
Abnormal	7 (7.6%)	8 (9.9%)		6 (8.2%)	7 (10.9%)	
ADC (×10^-3^mm^2^/s) ^a^	0.98 ± 0.26	1.13 ± 0.34	0.001^*^	1.00 ± 0.28	1.12 ± 0.34	0.023^*^
MD (cm) ^a^	8.72 ± 4.21	7.03 ± 4.28	0.001^*^	8.95 ± 4.42	7.31 ± 4.41	0.032^*^
SI on T2WI ^b^			0.002^*^			0.003^*^
Hypo-intensity	5 (5.4%)	19 (23.5%)		2 (2.7%)	14 (21.9%)	
Iso-intensity	40 (43.5%)	20 (24.7%)		34 (46.6%)	17 (26.6%)	
Hyperintensity	30 (32.6%)	23 (28.4%)		23 (31.5%)	17 (26.6%)	
Mixed	11 (12%)	15 (18.5%)		9 (12.3%)	13 (20.3%)	
Solid and cystic components ^b^			0.004^*^			0.002^*^
Predominantly cystic	30 (32.6%)	12 (14.8%)		26 (35.6%)	10 (15.6%)	
Cystic-solid	21 (22.8%)	13 (16.1%)		17 (23.3%)	9 (14.1%)	
Predominantly solid	41 (44.6%)	56 (69.1%)		30 (41.4%)	45 (70.3%)	
Hemorrhage ^b^			0.188			0.154
Present	16 (17.4%)	8 (9.9%)		13 (17.8%)	6 (9.4%)	
Absent	76 (82.6%)	73 (90.1%)		60 (82.2%)	58 (90.6%)	

EOC, epithelial ovarian cancer; SCST, sex cord-stromal tumors; ROMA, Risk of Ovarian Malignancy Algorithm; CA125, Cancer antigen 125; ADC, apparent diffusion coefficient; MD, maximum diameter; SI, signal intensity; T2WI, T2 weighted imaging; ^a^ Data are the mean ± standard deviation, P values calculated by sample t test. ^b^ Date are the case (%), P values calculated by Chi-square tests or Fisher exact test. * P values< 0.05 were considered statistically significant.

### Feature extraction and selection

**Figure 3:**
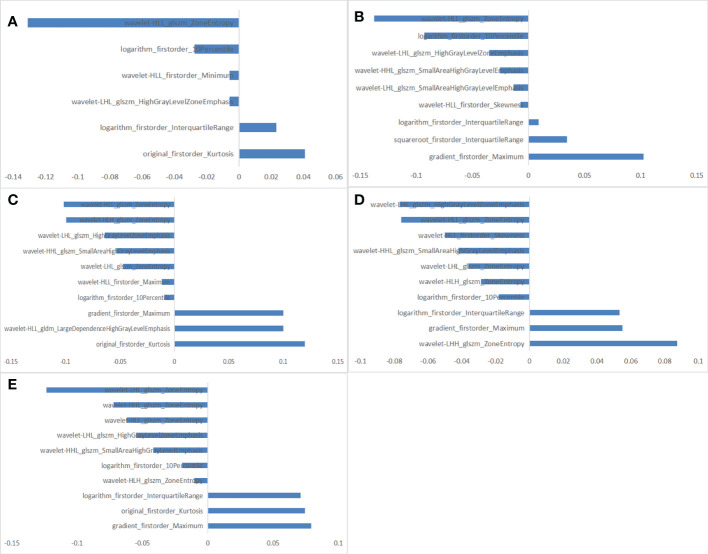
The selected radiomic features in each fold. **(A)** 6 radiomic features selected in fold 1. **(B)** 9 radiomic features selected in fold 2. **(C)** 10 radiomic features selected in fold 3. **(D)** 10 radiomic features selected in fold 4. **(E)** 10 radiomic features selected in fold 5.

Of all the extracted radiomics features, the median ICC was 0.90, and 1277 of 1409 features (91%) were robust and were selected for subsequent analysis, with ICC > 0.75. Lassolars algorithms on feature selection for each fold are shown in **Figure S1**. The selected radiomic features and the corresponding coefficients in each fold are shown in **Figure 3** and **Table S2**. We did model building in every fold, the AUCs for the 5-fold cross validation are reported in **Table S3** and **Figure S2**, and the mean AUCs were 0.883 ± 0.018 in the training cohort and 0.849 ± 0.021 in the validation cohort. Finally, there were three features got selected in 3 of the 5 folds, three features got selected in 4 of the 5 folds, and three features got selected in every fold. The frequencies of the radiomics features are summarized in **Table 3**.

**Table 3:** Details of the selected features in the developed radiomics models.

Number	Radiomics feature name	Frequency
1	wavelet-HLL-GLSZM-ZoneEntropy	5
2	wavelet-LHL-GLSZM-HighGrayLevelZoneEmphasis	5
3	logarithm-firstorder-10Percentile	5
4	logarithm-firstorder-InterquartileRange	4
5	gradient-firstorder-Maximum	4
6	wavelet-HL-GLSZM-SmallAreaHighGrayLevelEmphasis	4
7	original-firstorder-Kurtosis	3
8	wavelet-HLH- GLSZM -ZoneEntropy	3
9	wavelet-LHL- GLSZM -ZoneEntropy	3

GLSZM, Gray-Level Size Zone Matrix

### Construction and performance of the prediction models

**Figure 4:**
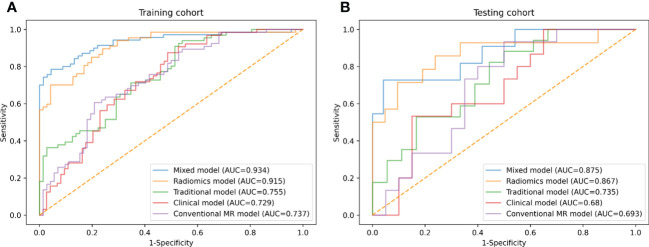
ROC curves of conventional MR model, clinical model, traditional model, radiomics model and mixed model within the development **(A)** and testing **(B)** cohorts.

Based on the selected clinical variable ROMA index, a clinical model was established. Based on parameter ADC, solid and cystic components, a conventional MR model was established. Then, based on the combination of the above clinical factors and conventional MR parameters, a traditional model was established. The AUCs of the clinical model, conventional MR model, and traditional model were 0.729 (95%CI: 0.645-0.812), 0.737 (95%CI: 0.654-0.819), 0.755 (95%CI: 0.677-0.834) in the development cohort, respectively, and 0.680 (95%CI: 0.498-0.862), 0.693 (95%CI: 0.515-0.872), 0.735 (95%CI: 0.569-0.902) in the testing cohort, respectively (**Figure 4** and **Table 4**).

**Table 4:** Predictive performances of the final models in the development and testing cohorts.

Model	Development cohort					Testing cohort				
	AUC (95% CI)	SEN	SPE	ACC	*P* value	AUC (95% CI)	SEN	SPE	ACC	*P* value
Conventional MR model	0.737 (0.654-0.819)	0.758	0.528	0.638	<0.001	0.693 (0.515-0.872)	0.600	0.650	0.629	0.039
Clinical model	0.729 (0.645-0.812)	0.812	0.541	0.667	<0.001	0.680 (0.498-0.862)	0.600	0.650	0.629	0.048
Traditional model	0.755 (0.677-0.834)	0.727	0.542	0.630	<0.001	0.735 (0.569-0.902)	0.706	0.611	0.657	0.017
Radiomics model	0.915 0.869-0.962)	0.701	0.873	0.790	<0.001	0.867 (0.732-1.000)	0.714	0.857	0.800	<0.001
Mixed model	0.934 (0.892-0.976)	0.814	0.868	0.841	<0.001	0.875 (0.743-1.000)	0.727	0.750	0.743	<0.001

AUC, area under curve; SEN, sensitivity; SPE, speciﬁcity; ACC, accuracy; p values on the AUCs are difference from AUC=0.5

Three different radiomics models (Models 1-3) were developed using the following combinations of the radiomics features: 3 features (got selected in every fold), 6 features (got selected 4 or more times repeatedly) and 9 features (got selected 3 or more times repeatedly). Among them, Model 3 showed the best performance, which was determined as the final radiomics model, with the AUCs of 0.915 (95% CI: 0.869-0.962) and 0.867 (95% CI: 0.732-1.000) in the development and testing cohorts, respectively (**Figure 4** and **Table 4**). However, no significant differences were observed between Model 3 and the other two models. The AUCs of the three models and the comparisons in terms of diagnostic performance among them were shown in **Table 5**. Rad-score based on 9 features were weighted by their respective coefficients, the calculation formula for the Rad-score is provided in the **Supplementary Materials** page 7.

**Table 5:** Predictive performances of the three developed radiomics models.

Model	Development cohort					Testing cohort				
	AUC (95% CI)	SEN	SPE	ACC	*P* value	AUC (95% CI)	SEN	SPE	ACC	*P* value
Model 1	0.837 (0.765-0.909)	0.672	0.838	0.761	0.073	0.817 (0.662-0.971)	0.800	0.55	0.657	0.631
Model 2	0.873 (0.814-0.932)	0.683	0.853	0.775	0.269	0.846 (0.707-0.985)	0.769	0.818	0.800	0.832
Model 3	0.915 (0.869-0.962)	0.701	0.873	0.790	1.000	0.867 (0.732-1.000)	0.714	0.857	0.800	1.000

AUC, area under curve; SEN, sensitivity; SPE, speciﬁcity; ACC, accuracy; P values calculated by Delong test, compared with Model 3.

Finally, we established a mixed model based on the Rad-score, clinical characteristics (ROMA), and conventional MR parameters (ADC, solid and cystic components). The AUCs of the mixed model were 0.934 (95% CI: 0.892-0.976) and 0.875 (95% CI: 0.743-1.000) in the development and testing cohorts, respectively (**Figure 4** and **Table 4**).

The difference between the traditional model and radiomics model was statistically significant in the testing cohort (traditional model AUC *vs*. radiomics model AUC: 0.735 *vs*. 0.867, Delong test *P*< 0.001); however, there was no evidence of a difference in the testing set between the radiomics model compared with the mixed model (radiomics model AUC *vs*. mixed model AUC: 0.867 *vs*. 0.875, Delong test *P* = 0.561).

**Figure 5:**
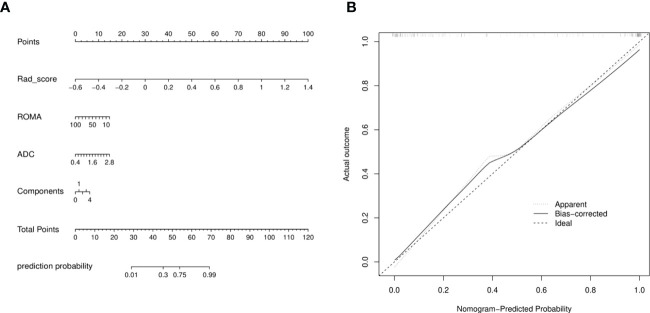
**(A)** Radiomics nomogram with Rad-score, two conventional MR findings and a clinical factor, including components, ADC, and ROMA. **(B)** Calibration curves of radiomics nomogram. The diagonal line represented the perfect prediction of the radiomics nomogram. The black solid line represented the calibration curve of nomogram in the testing cohorts. The calibration curves were close to the diagonal line, which indicated good prediction performance of the nomogram.

A radiomics nomogram was constructed by using the selected variables from multivariate logistic regression and Rad-score to provide a visualized outcome measure (**Figure 5A**). The total score for this nomogram was calculated using the formula: Nomo-score = -1.5402 + 11.4118 × Rad-score -0.0335 × ROMA + 1.3928 × ADC + 0.3532 × Components. The calibration curves demonstrated good diagnostic consistency between the predictions of the radiomics nomogram and the actual observations of the samples (**Figure 5B**).

**Figure 6:**
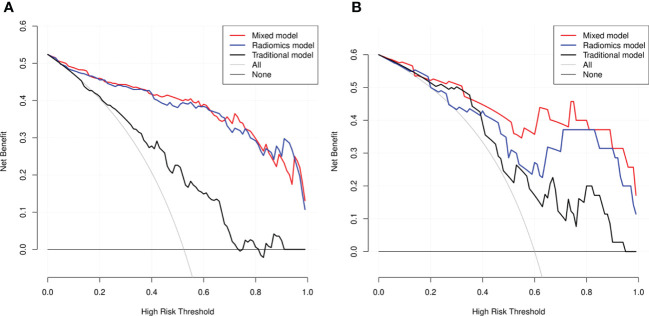
Decision curve analysis (DCA) for the three models within the development **(A)** and testing **(B)** cohorts. The net benefit versus the threshold probability was plotted. The x-axis represented the threshold probability, while the y-axis represented the net benefits. The sensitivity and specificity of the model were calculated at each threshold to determine the net benefit. The DCAs showed that the net benefits of the mixed model (black line) and the radiomics model (red line) were superior to the benefits of the traditional model (blue line) with the threshold probability range from 0 to 1.

DCA revealed that the radiomics model and the mixed model provided a better net benefit than the traditional model across the majority of the range of reasonable threshold probabilities (**Figure 6**).

## Discussion

SCSTs are composed of a heterogeneous group with complicated image manifestation. Although a few cases are mainly cystic, most SCSTs are predominantly solid masses (2, 4, 5). In MRI, the presence of solid tissue in an adnexal lesion is the primary cause of increased risk stratification (7, 14), so the particular lesion that requires to be differentiated is EOC. With the optimization of various diagnostic schemes, multiple methods have been gradually combined to evaluate adnexal tumors (15). In the current study, we assessed the ability of the clinical model, conventional MR model, traditional model, and the radiomics model to distinguish SCSTs and EOCs. As shown in the result, radiomics model yielded prominent predictive performance, significantly higher than that of the traditional model. The mixed model stood out among all the models.

Generally, SCSTs are considered to be clinically different from epithelial tumors to some extent, they occur across a wide age range and some patients may have clinical signs of hormone production (5, 16). In our study, only 8 in 73 patients with SCSTs showed elevated hormone levels, mainly presenting as a slight increase in prolactin. No significant difference was observed in menstrual status or endocrine level between the two groups. In previous studies (17, 18), serum CA125 and ROMA index have been proven to be effective diagnostic markers for EOCs, which could be used to differentiate between benign and malignant lesions. The present results were consistent with the previous studies, revealing that serum CA125 and ROMA index in the SCSTs group were significantly lower than those in EOCs, owing to the blunt nature of SCSTs. And in the multivariate logistic regression analysis, ROMA index was an independent predictor. However, the diagnostic efficacy of the clinical model was not satisfactory (AUCs = 0.729 and 0.680 in the development and testing cohorts, respectively), suggesting that clinical characteristics can only provide limited information for the differential diagnosis.

Conventional MR images could provide abundant information regarding the pathological features of tumors. Yin et al. (19) reported their results of 36 thecomas/fibrothecomas and 40 malignant pelvic solid tumors *via* conventional MRI and DWI examinations. They found that signal intensity on T2WI, capsule, and the lowest ADC value were important indicators in discriminating thecomas/fibrothecomas from malignant pelvic solid tumors. Their study lacked other types of sex cord tumors, and they didn’t build predictive models. In our study, ADC value, solid and cystic components were independent predictors for differentiating EOCs and SCSTs. The ADC value of SCSTs was relatively higher than that of EOCs, and SCSTs were more likely to be dominated by solid masses. Conventional MR model showed that the discrimination ability of MR parameters was also limited. Moreover, after negotiation, most conventional MR parameters required subjective interpretation by the radiologist, resulting in a poor inter-observer agreement in the assessment of these features.

In this study, we used different combinations of the selected radiomics features to establish three radiomics models, all of which achieved good prediction efficiency. When combining the most efficient group of radiomics features and the traditional parameters, we found that the diagnostic performance of the final radiomics model was comparable to that of the mixed model. To date, in a few studies, radiomics have been applied to patients with adnexal tumors, such as evaluating the ability of texture features to characterize the histopathological classification of ovarian cancer (9–11) and to predict prognosis (8, 9, 20). However, SCSTs have been rarely discussed. To our knowledge, this study was the first one to establish an MR-based radiomics model focusing on the differentiation of SCSTs from others. The results are encouraging, and show great potential to improve the prediction accuracy for ovarian tumors and derive predictive imaging biomarkers. Misdiagnosis could thus be further avoided.

Radiomics extracts high-throughput quantitative data from medical images, which is helpful for disease diagnosis, staging, management, and prognostication. In the current study, we ran a 5-fold cross validation setup during the feature selection, where each fold we did feature selection and model building to provide stable features. Nine radiomics features were finally selected, mainly including first-order statistical features and Gray Level Size Zone Matrix (GLSZM). The features contain a variety of traceable image information. First-order statistics are based on histograms of the original image and describe the distribution of voxel intensities within the image region (21, 22). The GLSZM features quantify the Second-Order joint probabilities of images (21). For example, Zone Entropy (ZE) of GLSZM measures the randomness in the distribution of zone sizes and gray levels, a higher value indicates more heterogeneity in the texture patterns; High Gray Level Zone Emphasis of GLSZM measures the distribution of the higher gray-level values, with a higher value indicating a greater proportion of higher gray-level values and size zones in the image. In our study, GLSZM is a quite important set of features for differentiating SCSTs and EOCs, the intrinsic definition of these features imply that they may capture the presence of necrotic, edematous or cellular regions within the tumors (22).

To date, there is no consensus on which or how many MRI sequences should be used to establish a radiomics model. Some studies (11, 15, 23) have included several individual sequences at the same time or combined multiple sequences to establish a radiomics model for evaluation, suggesting that the multi-sequence combination model may have better performance. Only FS-T2WI sequences were used in our radiomic analysis, considering that FS-T2WI is an important and common sequence in the conventional pelvic MRI scanning protocol with the highest spatial resolution which would improve the visualization of ROIs. Sufficient predictive performance has been achieved for differentiating SCSTs and EOCs. Further research with more sequences such as DWI, ADC, and T1WI+C, would be carried out to improve our radiomics classifier.

Several limitations should be noted. First, for a radiomics study, the sample size was relatively small, so the results might be biased. Second, this study is a single-center study without external verification, so the reproducibility and generalizability of the models need to be further tested. In the future, a multicenter study with a larger dataset size should be conducted to perform an optimal radiomics analysis. Third, the majority of patients in the EOC group were in advanced stages, resulting in a demonstrated advantage of the radiomics model. Future research should include more indeterminate adnexal masses.

In conclusion, by comparing various models, we found the MR-based radiomics model achieved excellent prediction performance for differentiating SCSTs and EOCs. The mixed model which combining the radiomics features and traditional parameters achieve a performance comparable to the radiomics model. Therefore, we believe that the radiomics approach could be a more objective and accurate way for discriminating SCSTs and EOCs. Meanwhile, the mixed model developed in our study could provide a comprehensive, effective manner for clinicians to diagnose and develop appropriate management strategies.

## Data availability statement

The datasets presented in this article are not readily available because of institutional restriction. Requests to access the datasets should be directed to Xin Zhao, E-mail:zhaoxinxct@vip.163.com.

## Ethics statement

The studies involving human participants were reviewed and approved by The Third Affiliated Hospital of Zhengzhou University. Written informed consent for participation was not required for this study in accordance with the national legislation and the institutional requirements.

## Author contributions

Conception and design: MC. Administrative support: XZ. Provision of study materials or patients: ST, ZZ, and TP. Collection and assembly of data: TR, LZ, ZY, and LM. Data analysis and interpretation: MC and XY. Writing - review and editing: MC, ST, and KW. Final approval of manuscript: All authors. All authors contributed to the article and approved the submitted version.
