# Supplementary material for: Magnetic resonance imaging radiomics to differentiate ovarian sex cord-stromal tumors and primary epithelial ovarian cancers
Source: Front Oncol. 2023 Jan 13;12:1073983. doi: 10.3389/fonc.2022.1073983 (PMC9880468; doi:10.3389/fonc.2022.1073983)
Supplement: Supplementary file 1 [file DataSheet_1.docx]

**Figures**


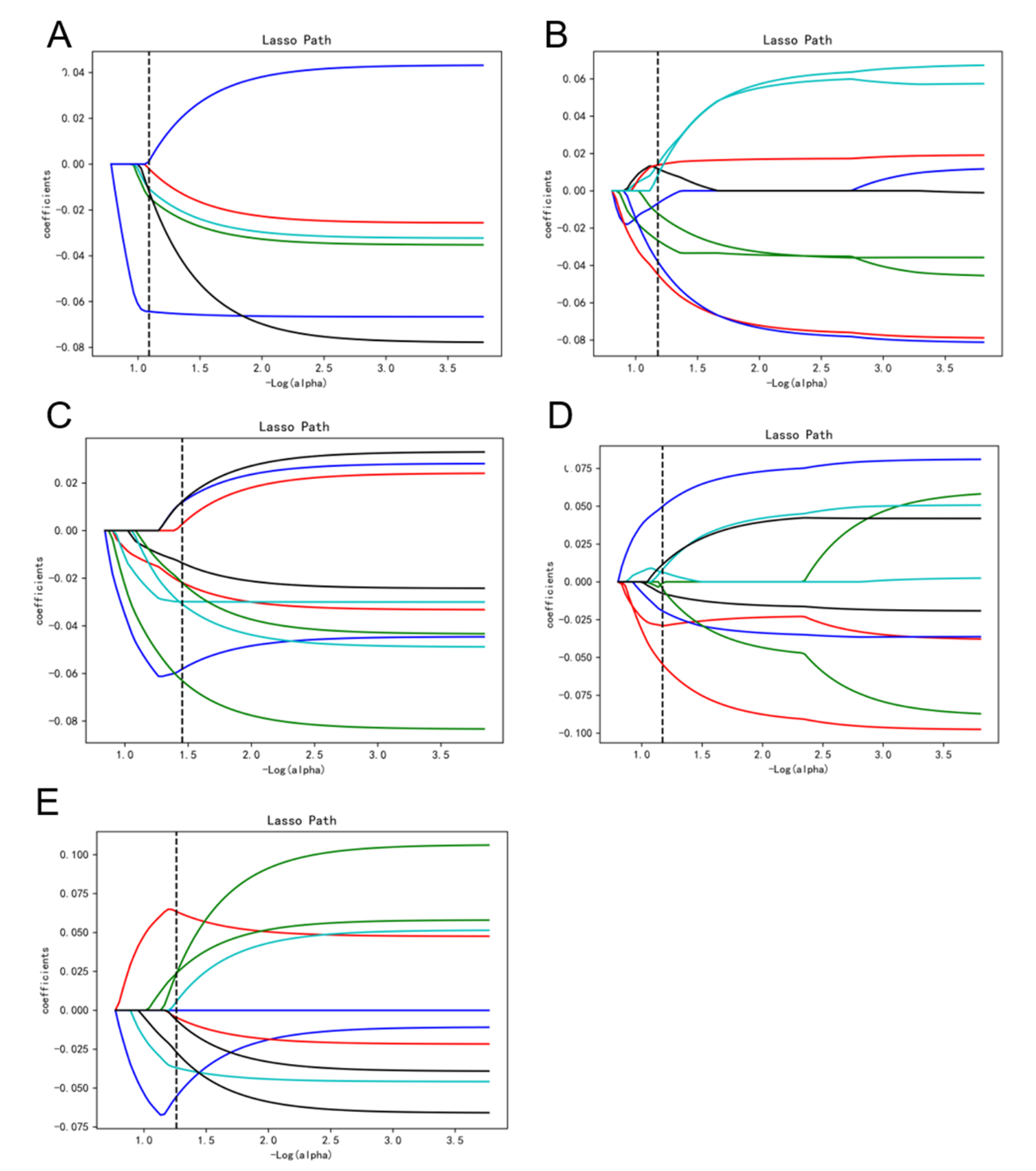


**Figure S1** Lassolars algorithms on feature selection for each fold. (A) Fold 1. (B) Fold 2. (C) Fold 3. (D) Fold 4. (E) Fold 5


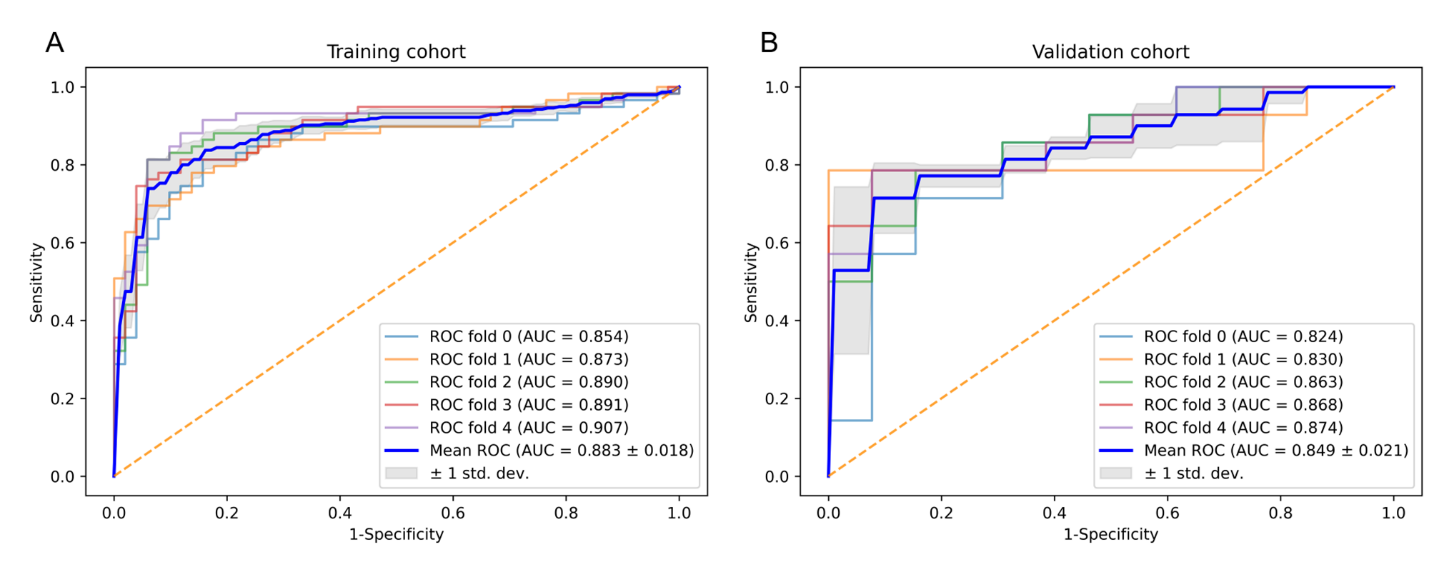


**Figure S2** ROCs for the 5-fold cross validation in the training cohort (A) and validation cohort (B).

**Tables**

| **Table S1 Representative scanning sequences and parameters** | | | | | | |
| --- | --- | --- | --- | --- | --- | --- |
| **Sequence** | **TR/TE（msec）** | **FOV**  **(cm²)** | **Matrix** | **Slice thickness (mm)** | **Flip Angle**  **(degree)** | **NEX** |
| 3.0T signa pioneer, GE Healthcare | | | | | | |
| Axial T1WI | 464/7.01 | 32×36.4 | 256×320 | 5/6 | 111 | 1 |
| Axial T2WI | 5518/83.2 | 30×34.1 | 288×288 | 5/6 | 110 | 2 |
| Sagittal T2WI | 5938/83.10 | 24×27.3 | 288×288 | 4/5 | 111 | 2 |
| Coronal T2WI | 4811/82.62 | 30×34.1 | 244×320 | 5/6 | 111 | 1 |
| DWI | 4000/75.2 | 34×38.6 | 128×128 | 5/6 | 90 | 6 |
|  | b=0, 1000 s/mm2, ADC map was automatically generated by post process procedure | | | | | |
| DCE-MRI | 4.79/1.80 | 36×40.9 | 260×260 | 1.4/1.4 | 15 | 0.7 |
| Multiphasic 3D gradient echo sequence（3D-LAVA）:Intravenous injection of 0.1mmol/kg Gd-DTPA, scanning 19 phases (13 seconds for each phase) | | | | | | |
| 3.0-T Skyra, Siemens Healthcare | | | | | | |
| Axial T1WI | 547/19 | 24×27.1 | 288×384 | 4/5 | 120 | 1 |
| Axial T2WI | 3500/85 | 30×34.1 | 288×384 | 4/5 | 120 | 1 |
| Sagittal T2WI | 2520/99 | 24×27.3 | 320×320 | 5/6 | 140 | 1 |
| Coronal T2WI | 5410/85 | 30×34.1 | 288×384 | 5/6 | 120 | 1 |
| DWI | 5100/51 | 38×43.2 | 95×160 | 4/5 | 90 | 1 |
|  | b=0, 500, 800 s/mm2, ADC map was automatically generated by post process procedure | | | | | |
| DCE-MRI | 3.31/1.30 | 38×43.2 | 195×320 | 3/- | 9 | 1 |
| Multiphasic 3D gradient echo sequence（t1_vibe_fs_ce）:40, 60, 80, and 120s after the intravenous injection of 0.1 mmol/kg Gd-DTPA | | | | | | |

T1WI, T1 weighted imaging; T2WI, T2 weighted imaging; DWI, diffusion-weighted imaging; ADC, apparent diffusion coefficient; DCE, dynamic contrast enhanced

| **Table S2 The selected radiomic features and the corresponding coefficients in each fold** | | |
| --- | --- | --- |
|  | **Radiomics feature name** | **coefficients** |
| **Fold 1** | original_firstorder_Kurtosis | 0.0409 |
|  | logarithm_firstorder_InterquartileRange | 0.02316 |
|  | wavelet-LHL_GLSZM_HighGrayLevelZoneEmphasis | -0.00595 |
|  | wavelet-HLL_firstorder_Minimum | -0.00595 |
|  | logarithm_firstorder_10Percentile | -0.0277 |
|  | wavelet-HLL_GLSZM_ZoneEntropy | -0.13103 |
| **Fold 2** | gradient_firstorder_Maximum | 0.10254 |
|  | squareroot_firstorder_InterquartileRange | 0.03425 |
|  | logarithm_firstorder_InterquartileRange | 0.00905 |
|  | wavelet-HLL_firstorder_Skewness | -0.00725 |
|  | wavelet-LHL_GLSZM_SmallAreaHighGrayLevelEmphasis | -0.01348 |
|  | wavelet-HHL_GLSZM_SmallAreaHighGrayLevelEmphasis | -0.02562 |
|  | wavelet-LHL_GLSZM_HighGrayLevelZoneEmphasis | -0.03486 |
|  | logarithm_firstorder_10Percentile | -0.09295 |
|  | wavelet-HLL_GLSZM_ZoneEntropy | -0.13768 |
| **Fold 3** | original_firstorder_Kurtosis | 0.1198 |
|  | wavelet-HLL_GLDM_LargeDependenceHighGrayLevelEmphasis | 0.10013 |
|  | gradient_firstorder_Maximum | 0.10004 |
|  | logarithm_firstorder_10Percentile | -0.00902 |
|  | wavelet-HLL_firstorder_Maximum | -0.01139 |
|  | wavelet-LHL_GLSZM_ZoneEntropy | -0.04724 |
|  | wavelet-HHL_GLSZM_SmallAreaHighGrayLevelEmphasis | -0.05337 |
|  | wavelet-LHL_GLSZM_HighGrayLevelZoneEmphasis | -0.0638 |
|  | wavelet-HLH_GLSZM_ZoneEntropy | -0.09918 |
|  | wavelet-HLL_GLSZM_ZoneEntropy | -0.1014 |
| **Fold 4** | wavelet-LHH_GLSZM_ZoneEntropy | 0.08734 |
|  | gradient_firstorder_Maximum | 0.05486 |
|  | logarithm_firstorder_InterquartileRange | 0.05329 |
|  | logarithm_firstorder_10Percentile | -0.01821 |
|  | wavelet-HLH_GLSZM_ZoneEntropy | -0.02872 |
|  | wavelet-LHL_GLSZM_ZoneEntropy | -0.03633 |
|  | wavelet-HHL_GLSZM_SmallAreaHighGrayLevelEmphasis | -0.04203 |
|  | wavelet-HLL_firstorder_Skewness | -0.05009 |
|  | wavelet-HLL_GLSZM_ZoneEntropy | -0.076 |
|  | wavelet-LHL_GLSZM_HighGrayLevelZoneEmphasis | -0.07685 |
| **Fold 5** | gradient_firstorder_Maximum | 0.07923 |
|  | original_firstorder_Kurtosis | 0.07449 |
|  | logarithm_firstorder_InterquartileRange | 0.07108 |
|  | wavelet-HLH_GLSZM_ZoneEntropy | -0.01036 |
|  | logarithm_firstorder_10Percentile | -0.0197 |
|  | wavelet-HHL_GLSZM_SmallAreaHighGrayLevelEmphasis | -0.04166 |
|  | wavelet-LHL_GLSZM_HighGrayLevelZoneEmphasis | -0.05481 |
|  | wavelet-HLL_GLSZM_ZoneEntropy | -0.06213 |
|  | wavelet-HHL_GLSZM_ZoneEntropy | -0.07228 |
|  | wavelet-LHL_GLSZM_ZoneEntropy | -0.12368 |

GLSZM, Gray-Level Size Zone Matrix; GLDM, Gray Level Dependence Matrix

| **Table S3 Results of 5 folds cross validation based on development data** | | | | | | | | | |  |  |  |
| --- | --- | --- | --- | --- | --- | --- | --- | --- | --- | --- | --- | --- |
| **Fold** | **Training cohort** | | | |  | **Validation cohort** | | | | |  |  |
|  | **AUC (95%CI)** | **SEN** | **SPE** | **ACC** |  | **AUC (95%CI)** | **SEN** | **SPE** | **ACC** | | |  |
| Fold 1 | 0.854 (0.778-0.930) | 0.746 | 0.843 | 0.791 |  | 0.824 (0.660-0.989) | 0.714 | 0.846 | 0.778 | | |  |
| Fold 2 | 0.873 (0.805-0.940) | 0.729 | 0.863 | 0.791 |  | 0.830 (0.646-1.000) | 0.786 | 1.00 | 0.889 | | |  |
| Fold 3 | 0.890 (0.824-0.957) | 0.847 | 0.843 | 0.845 |  | 0.863 (0.724-1.000) | 0.857 | 0.692 | 0.778 | | |  |
| Fold 4 | 0.891 (0.827-0.955) | 0.814 | 0.882 | 0.845 |  | 0.868 (0.726-1.000) | 0.786 | 0.692 | 0.741 | | |  |
| Fold 5 | 0.907 (0.844-0.970) | 0.847 | 0.882 | 0.864 |  | 0.874 (0.740-1.000) | 0.786 | 0.923 | 0.852 | | |  |
| Mean AUC | 0.883±0.018 |  |  |  |  | 0.849±0.021 |  |  |  | | |  |
| AUC, area under curve; SEN, sensitivity; SPE, speciﬁcity; ACC, accuracy | | | | | | | | | |  |  |  |

**formula**

Rad-score=6.3375-0.9827×wavelet-HLL_glszm_ZoneEntropy

-1.0279×wavelet-LHL_glszm_HighGrayLevelZoneEmphasis

-0.4079×logarithm_firstorder_10Percentile

+0.0588×logarithm_firstorder_InterquartileRange

+0.9594×gradient_firstorder_Maximum

-0.6630×wavelet-HHL_glszm_SmallAreaHighGrayLevelEmphasis

-0.5527×wavelet-HLH_glszm_ZoneEntropy

-0.4614×wavelet-HLL_firstorder_Skewness

-0.2189×wavelet-LHL_glszm_ZoneEntropy
